# Supplementary material for: Learning to operate an imagined speech Brain-Computer Interface involves the spatial and frequency tuning of neural activity
Source: Commun Biol. 2025 Feb 20;8:271. doi: 10.1038/s42003-025-07464-7 (PMC11842755; doi:10.1038/s42003-025-07464-7)
Supplement: Supplementary file 1 — Supplementary Information [file 42003_2025_7464_MOESM1_ESM.pdf]

Supplementary Information

Supplementary Method

Experiment with discontinuous real-time feedback

During the *offline session*, participants imagined pronouncing one of the two syllables (/fɔ/ and /gi/) without receiving any real-time visual feedback. At the beginning of the trial, a written cue indicating which of the two syllables participants had to imagine pronouncing was presented inside a black circle, whose border was of the color associated with the cued syllable, respectively pink for /fɔ/ and blu for /gi/ (1 s, Supplementary Figure 1a). Participants were instructed to start the imagery right after the written cue disappeared and continue for 5 s while watching the filling of a circle turn progressively from black into one of two target colors. After the imagery period, participants had a rest period of 3 seconds before the next trial began. There were a total of 45 trials per syllable, arranged in 3 blocks (15 trials per syllable per block).

The *classifier calibration* was performed with the same method as described for the main experiment.

During the *Online BCI-control,* participants were asked to reach the target color associated with the cued syllable by performing the same imagery task as during the *offline* session. This time participants were told the color of the circle was driven by their brain signals in real-time, although this was only partially the case. The feedback was not constantly updated but only when the classifier output corresponded to the cued syllable, otherwise it remained unchanged. This way participants had the feeling of progressing throughout the session but were not systematically informed about their errors. Importantly, different from the main experiment, a relative change in the classifier output towards the cued syllable did not systematically trigger a change in the feedback but was based only on the classifier's output sign, which substantially decreased the feedback update rate.

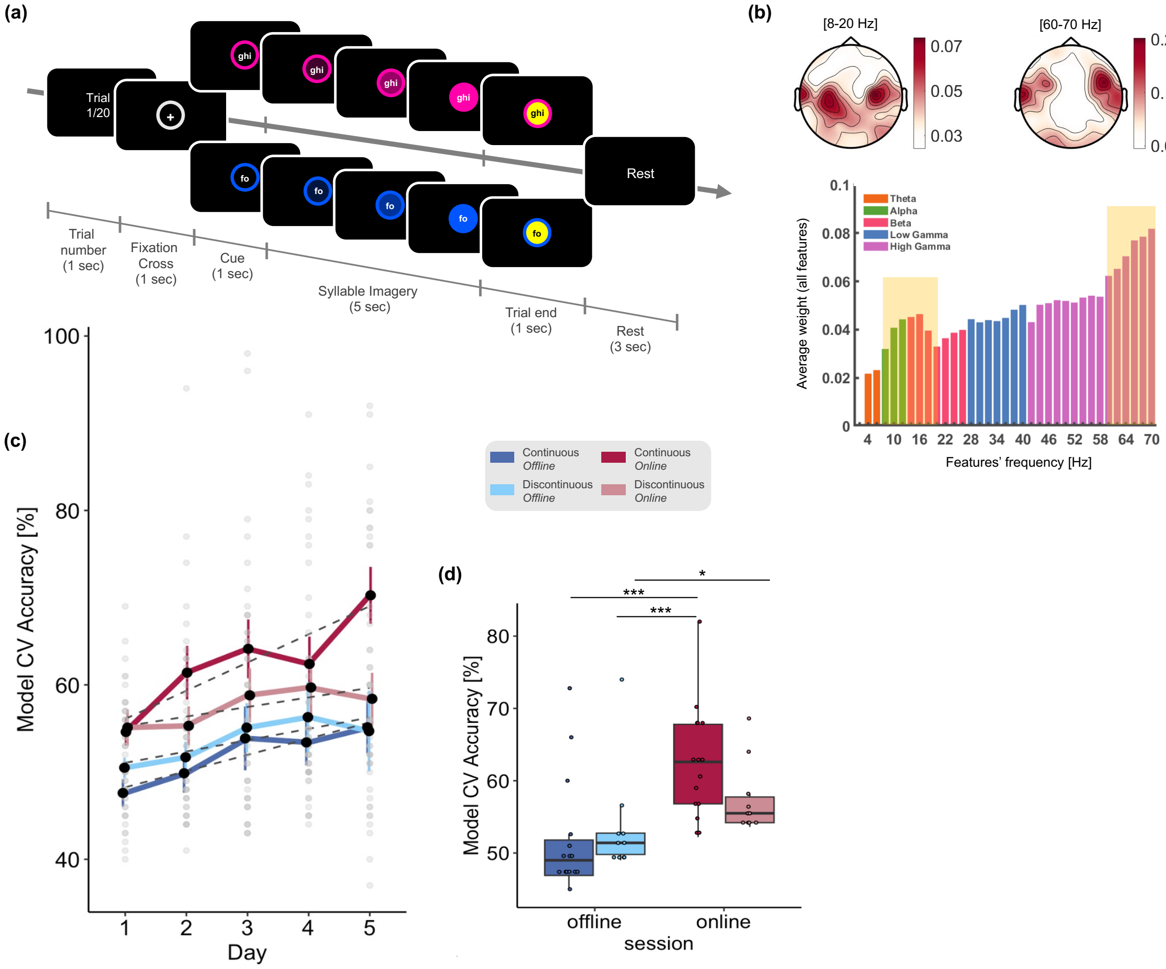

Supplementary Figure 1. Training with discontinuous feedback. (a) Experimental paradigm used by the group of participants who trained with the discontinuous feedback. **(b)** Decoding features: bar plots represent the average of features' weights for each frequency across all experimental days. Topographies of the average weights for these two frequency intervals are highlighted in the bar plot. (**c-d**) Cross-validation accuracy was obtained by computing the classifier in the *offline* (blue, cyan) and *online* (red, pink) sessions on each day for the continuous (darker colors, n = 15) and discontinuous feedback (lighter colors, n = 10). Error bars in (c) indicate the standard error of the mean. Boxes in (d) represent the interquartile range (IQR), with the horizontal line indicating the median, and whiskers extending to data points that are within 1.5× the IQR from the upper and lower quartile. Individual points represent data from a single participant. Significance is denoted with * for $p < 0.05$, and *** for $p < 0.001$.

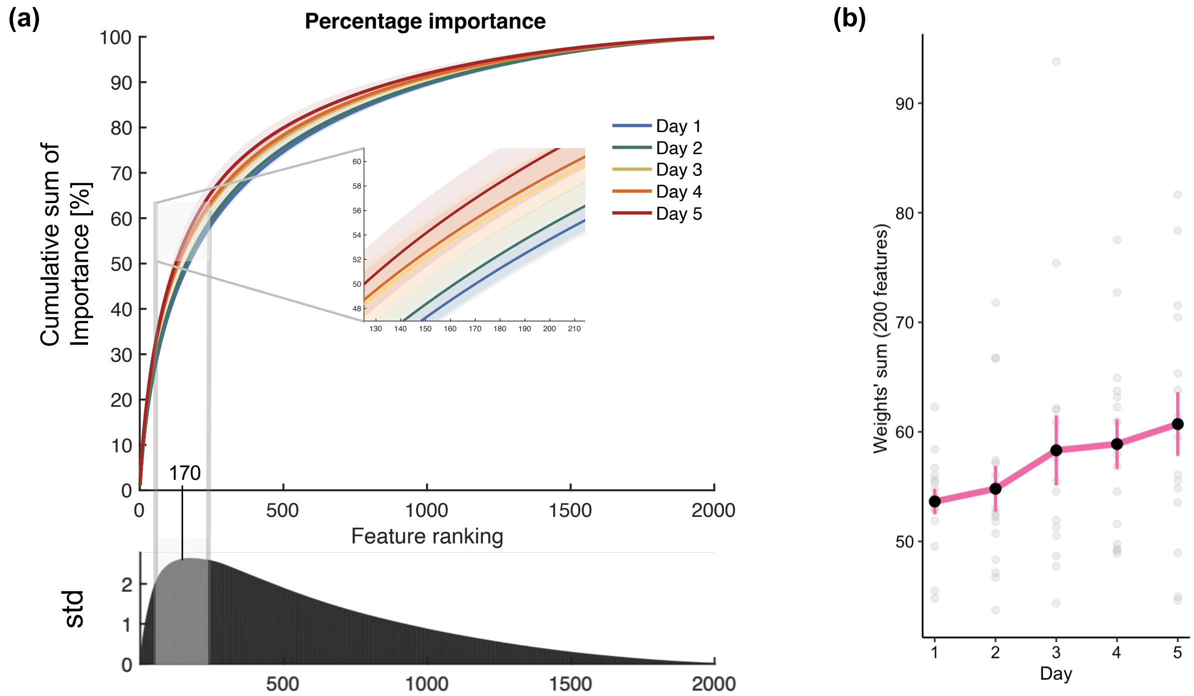

Supplementary Figure 2. Analysis of the classifier's features. The Random Forest classifier computes a weight for each feature (i.e. a channel-frequency pair), expressed as a percentage indicating how much the feature contributes to the accuracy of the model. The features are ranked according to their weight in descending order, from the highest to the lowest percentage. **(a)** Cumulative sum of the weights of the features along the ranking for each of the 5 days of training. The cumulative sum increases linearly from day 1 to day 5 so that as training progresses, a lower number of features is necessary to account for the same percentage. The value of 50% of cumulative importance is reached within the first 200 features across the 5 training days. The bottom plot illustrates, for each ranking position, the standard deviation calculated across the cumulative sums of the 5 training days. The variability increases up to the 170th ranking place, indicating that changes across training concentrate in a subset of most discriminant features and that features with lower ranking carry little information about changes over the 5 days. **(b)** Sum of the first 200 features' weight across all participants and each training day shows a linear increase. Error bars indicate the standard error of the mean.

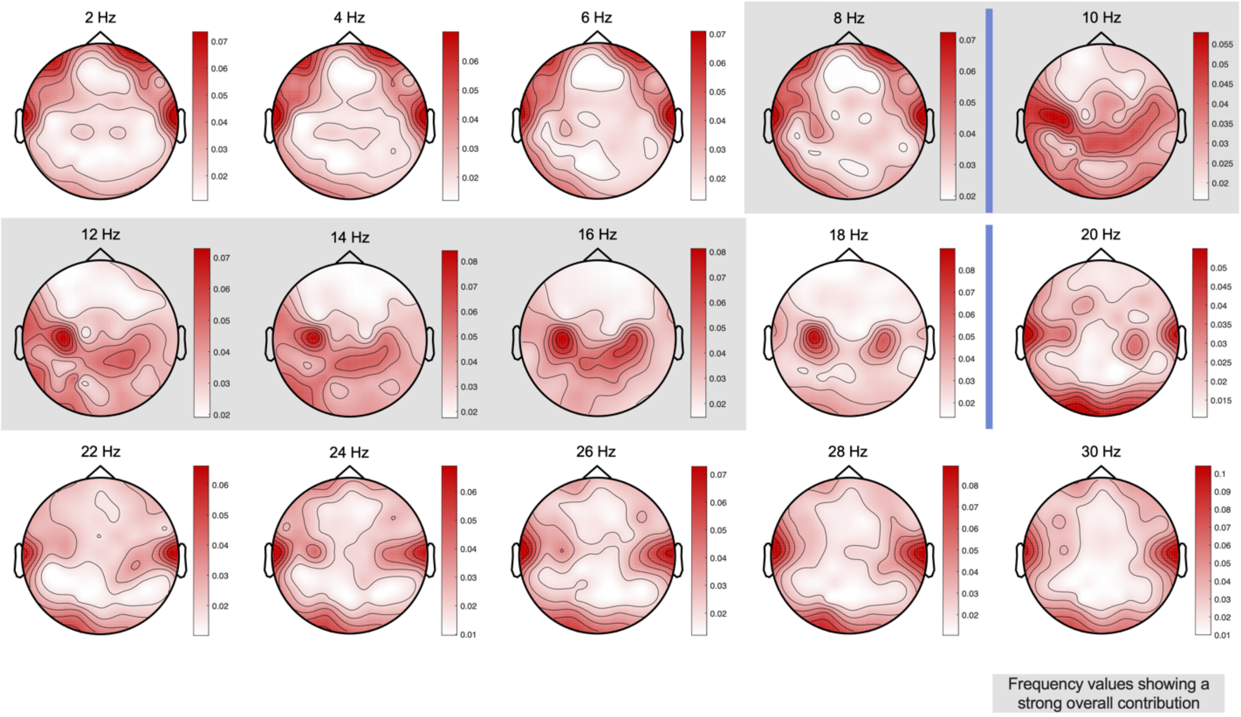

Supplementary Figure 3. Topographies of features' weight. Each topography represents the average features' weight at a certain frequency, from 2 Hz to 30 Hz. Average values are obtained by considering data from all participants and the 5 days of training. Vertical blue bars delimit topographical transitions, whereas grey backgrounds indicate topographies within one frequency range contributing the most to the discrimination between the two syllables (8-16 Hz, see Fig. 2c). Topographies beyond 30 Hz are not shown as strongly overlapping with the last one, at 30 Hz.

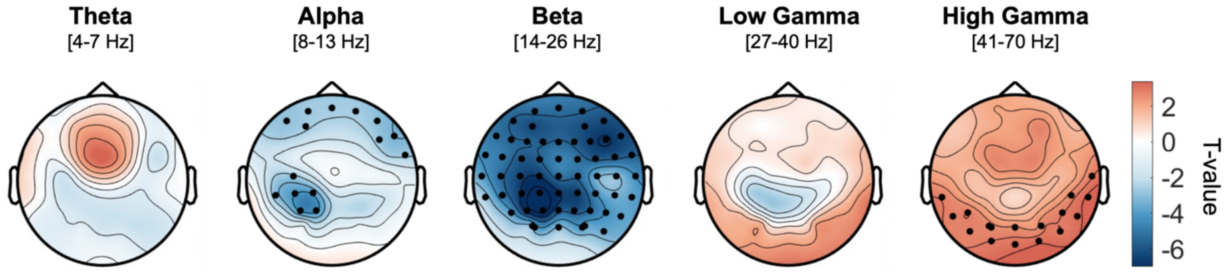

Supplementary Figure 4: **Power modulation during BCI-control.** EEG power modulation in the overall dataset (all participants and days) relative to the baseline (last second of the fixation cross). Power data averaged between 0 and 5 s for each frequency band were compared against the baseline using a within-participant paired t-test to find the channels showing significant power change (Cluster-based correction, two-tailed, target threshold α= 0.05, n = 15).

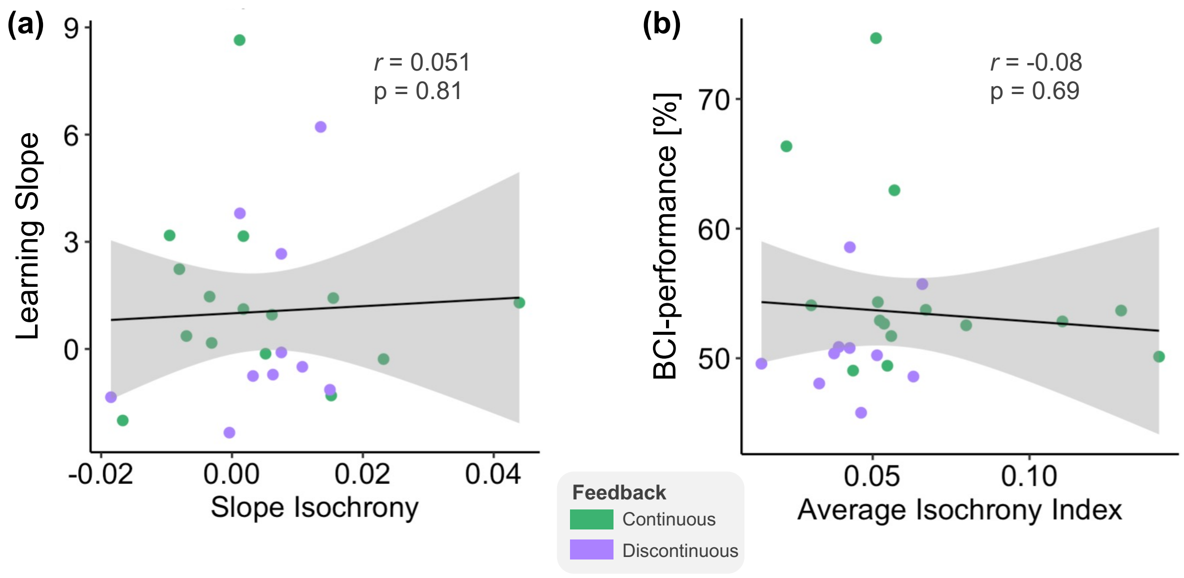

Supplementary Figure 5. Link between mental chronometry and behavioral results from BCI-control. (a) Correlation between the learning slope reflecting the change in BCI-performance across training and the slope obtained from the isochrony values (n = 25). **(b)** Correlation between the average BCI-performance and the average isochrony index both calculated considering the 5 days of training (n = 25).

Supplementary Table 1. Subjective reports of the strategy used to perform the BCI-control, from all volunteers who performed the 5-day of training with the continuous feedback. Reports are ordered according to the participant's learning slope, from highest (1) to lowest (15). Negative learning slopes are marked with * beside the ranking number.

Learning slope (↓)	Subjective report
1	Imagined articulation and pronouncing the syllable, good performance once made me overconfident and then lost the attention and control. Fatigued on one of the days so not so good control, but last day will full attention and good health was able to perform with very good control.
2	Thinking of the articulation, stress on both consonant and vowel, high focus, stressful during BCI control, focus more on offline training to perform better at online.
3	Imagined articulation, imagined articulation with visualizing syllables, lack of motivation affected performance, last day intensity of imagined articulation worked better.
4	Imagining articulation, focus on the vowel, faster rhythm.
5	Rhythmic repetition of each syllable, focus on the articulatory muscles (lips vs throat), for 'gi' used long rhythm and shouting in head, but for 'fo' repeated frequently to control.
6	Imagined articulation, intensity of the imagination increased by shouting in the head.
7	Imagined articulation and focus.
8	Imagined articulating the syllables with intense feeling of sensory activity.
9	Imagined articulation and developing it over the days, with training feeling much relaxed during the BCI control which helped in controlling, difficulty to switch between the syllables.
10	Imagined articulation, high attention, calmness and focus helped to improve performance over the days. Somewhat tired after all the sessions.
11	Imagined articulation, stopped breathing during the trials on 1st day so tried normal breathing on the other days, gets stressed when the bar goes down during online control.
12*	Imagined articulation, and increased intensity of articulation but did not work very well over the days. Rhythm of the repetition slowed down on purpose to see if BCI control could be achieved.
13*	Imagining articulation, strategy of learning new sounds, difficulty in switching between the two syllables during BCI control.
14*	Imagination of mouth movement, thinking of imagining syllables from the perspective of people wo cannot speak. Relaxation helped.
15*	Imagined articulation, found control with breathing, but not covert speech.<br>Imagining about the shape of mouth and teaching sounds to kids' strategy. Changing the rhythm and relaxing helped to have some control, fatigue deteriorated performance.
