# Supplementary material for: Learning to operate an imagined speech Brain-Computer Interface involves the spatial and frequency tuning of neural activity
Source: Commun Biol. 2025 Feb 20;8:271. doi: 10.1038/s42003-025-07464-7 (PMC11842755; doi:10.1038/s42003-025-07464-7)
Supplement: Supplementary file 2 — Reporting Summary [file 42003_2025_7464_MOESM2_ESM.pdf]

nature portfolio

Corresponding author(s): Silvia Marchesotti

Last updated by author(s): Dec 21, 2024

Reporting Summary

Nature Portfolio wishes to improve the reproducibility of the work that we publish. This form provides structure for consistency and transparency in reporting. For further information on Nature Portfolio policies, see our Editorial Policies and the Editorial Policy Checklist.

Statistics

For all statistical analyses, confirm that the following items are present in the figure legend, table legend, main text, or Methods section.

n/a	Confirmed	
☐	☒	The exact sample size (*n*) for each experimental group/condition, given as a discrete number and unit of measurement
☐	☒	A statement on whether measurements were taken from distinct samples or whether the same sample was measured repeatedly
☐	☒	The statistical test(s) used AND whether they are one- or two-sided<br>*Only common tests should be described solely by name; describe more complex techniques in the Methods section.*
☐	☒	A description of all covariates tested
☐	☒	A description of any assumptions or corrections, such as tests of normality and adjustment for multiple comparisons
☐	☒	A full description of the statistical parameters including central tendency (e.g. means) or other basic estimates (e.g. regression coefficient) AND variation (e.g. standard deviation) or associated estimates of uncertainty (e.g. confidence intervals)
☐	☒	For null hypothesis testing, the test statistic (e.g. *F*, *t*, *r*) with confidence intervals, effect sizes, degrees of freedom and *P* value noted<br>*Give P values as exact values whenever suitable.*
☒	☐	For Bayesian analysis, information on the choice of priors and Markov chain Monte Carlo settings
☒	☐	For hierarchical and complex designs, identification of the appropriate level for tests and full reporting of outcomes
☐	☒	Estimates of effect sizes (e.g. Cohen's *d*, Pearson's *r*), indicating how they were calculated

Our web collection on statistics for biologists contains articles on many of the points above.

Software and code

Policy information about availability of computer code

Data collection	Electroencephalography (EEG) and Electromyography (EMG) data were acquired using Lab Streaming Layer (LSL, https://github.com/sccn/labstreaminglayer).
Data analysis	The BCI loop used for the real-time analysis of the EEG data was developed using an adapted version of the framework Neurodecode (Fondation Campus Biotech Geneva, https://github.com/fcbg-hnp/NeuroDecode).<br>EEG data were preprocessed offline using Fieldtrip (Oostenveld et al., 2011) and the Semi Automatic Selection of Independent Component Analysis (SASICA, Chaumon et al., 2015) toolboxes within the MATLAB environment (version R2018b; The MathWorks, Natick, MA, USA).<br>All statistical analyses were carried out using MATLAB (version R2018b, The MathWorks, 331 Natick, MA, USA) and R version 4.1 (R Core Team, 2021).<br>Custom-made scripts for the analyses can be found at this link: https://osf.io/vr26k/.

For manuscripts utilizing custom algorithms or software that are central to the research but not yet described in published literature, software must be made available to editors and reviewers. We strongly encourage code deposition in a community repository (e.g. GitHub). See the Nature Portfolio guidelines for submitting code & software for further information.

Data

Policy information about availability of data

All manuscripts must include a data availability statement. This statement should provide the following information, where applicable:

- Accession codes, unique identifiers, or web links for publicly available datasets
- A description of any restrictions on data availability
- For clinical datasets or third party data, please ensure that the statement adheres to our policy

Source data underlying the graphs can be found at this link: https://osf.io/vr26k/. Raw data that support the findings of this study are available from the corresponding author upon reasonable request.

Human research participants

Policy information about studies involving human research participants and Sex and Gender in Research.

Reporting on sex and gender	Our main study included 5 females and 10 males, all healthy volunteers, while the control experiment included 5 females and 5 males. The gender is reported in the manuscript according to self-reporting and was not considered in the study design. We have no reason to assume that our results were confounded by this mild gender imbalance of our cohort. The task consisted in imagining pronouncing syllables, that to the best of our knowledge it is unlikely to elicit gender differences at the behavioral and neurophysiological level.
Population characteristics	Our study does not include any socially constructed or socially relevant categorization variable.
Recruitment	Participants were recruited through advertisement posted on the internet and word of mouth.
Ethics oversight	The study was approved by the local Ethics Committee (Commission cantonale d'éthique de la recherche, project 2022-00451).

Note that full information on the approval of the study protocol must also be provided in the manuscript.

Field-specific reporting

Please select the one below that is the best fit for your research. If you are not sure, read the appropriate sections before making your selection.

☒ Life sciences ☐ Behavioural & social sciences ☐ Ecological, evolutionary & environmental sciences

For a reference copy of the document with all sections, see nature.com/documents/nr-reporting-summary-flat.pdf

Life sciences study design

All studies must disclose on these points even when the disclosure is negative.

Sample size	We did not engage in any sample size calculation beforehand. Our sample size, in terms of number individual participants, aligns with that of most published BCI-EEG studies (also Mike X Cohen "Analyzing Neural Time Series Data"). Importantly, in our study, each participant took part in 5 distinct EEG recording sessions over 5 consecutive days. Consequently, we collected considerable amounts of data, corresponding to 12-13 hours of experimental time per participant.<br>Based on these reasons, we believe our sample size is appropriate to address our scientific questions.
Data exclusions	No data were excluded.
Replication	Our study relies upon measuring neurophysiological and behavioral changes across 5 days of training, that we expect to vary on an individual basis. We did not aim at replicating previous findings.
Randomization	Randomization was not relevant for the present study as the experimental conditions were the same for all participants.
Blinding	Blinding was not relevant for the present study (see above).

Reporting for specific materials, systems and methods

We require information from authors about some types of materials, experimental systems and methods used in many studies. Here, indicate whether each material, system or method listed is relevant to your study. If you are not sure if a list item applies to your research, read the appropriate section before selecting a response.

Materials & experimental systems

n/a	Involved in the study
☒	☐ Antibodies
☒	☐ Eukaryotic cell lines
☒	☐ Palaeontology and archaeology
☒	☐ Animals and other organisms
☒	☐ Clinical data
☒	☐ Dual use research of concern

Methods

n/a	Involved in the study
☒	☐ ChIP-seq
☒	☐ Flow cytometry
☒	☐ MRI-based neuroimaging
